# Effects of furosemide, acetazolamide and amiloride on renal cortical and medullary tissue oxygenation in non‐anaesthetised healthy sheep

**DOI:** 10.1113/EP091479

**Published:** 2024-03-29

**Authors:** Connie P. C. Ow, Nobuki Okazaki, Naoya Iguchi, Rachel M. Peiris, Roger G. Evans, Sally G. Hood, Clive N. May, Rinaldo Bellomo, Yugeesh R. Lankadeva

**Affiliations:** ^1^ Preclinical Critical Care Unit, Florey Institute of Neuroscience and Mental Health University of Melbourne Melbourne Victoria Australia; ^2^ Department of Anesthesiology and Resuscitology Okayama University Okayama Japan; ^3^ Department of Anesthesiology and Intensive Care Medicine Graduate School of Medicine Osaka University Osaka Japan; ^4^ Cardiovascular Disease Program, Biomedicine Discovery Institute and Department of Physiology Monash University Melbourne Victoria Australia; ^5^ Department of Critical Care, Melbourne Medical School University of Melbourne Melbourne Victoria Australia; ^6^ Australian and New Zealand Intensive Care Research Centre (ANZIC‐RC), School of Public Health and Preventive Medicine Monash University Melbourne Australia; ^7^ Department of Intensive Care Austin Hospital Melbourne Australia; ^8^ Department of Intensive Care Royal Melbourne Hospital Melbourne Australia; ^9^ Data Analytics Research and Evaluation Centre Austin Hospital Melbourne Australia

**Keywords:** acetazolamide, amiloride, furosemide, hypoxia, renal oxygenation

## Abstract

It has been proposed that diuretics can improve renal tissue oxygenation through inhibition of tubular sodium reabsorption and reduced metabolic demand. However, the impact of clinically used diuretic drugs on the renal cortical and medullary microcirculation is unclear. Therefore, we examined the effects of three commonly used diuretics, at clinically relevant doses, on renal cortical and medullary perfusion and oxygenation in non‐anaesthetised healthy sheep. Merino ewes received acetazolamide (250 mg; *n* = 9), furosemide (20 mg; *n* = 10) or amiloride (10 mg; *n* = 7) intravenously. Systemic and renal haemodynamics, renal cortical and medullary tissue perfusion and $P_{O_{2}}$, and renal function were then monitored for up to 8 h post‐treatment. The peak diuretic response occurred 2 h (99.4 ± 14.8 mL/h) after acetazolamide, at which stage cortical and medullary tissue perfusion and $P_{O_{2}}$ were not significantly different from their baseline levels. The peak diuretic response to furosemide occurred at 1 h (196.5 ± 12.3 mL/h) post‐treatment but there were no significant changes in cortical and medullary tissue oxygenation during this period. However, cortical tissue $P_{O_{2}}$ fell from 40.1 ± 3.8 mmHg at baseline to 17.2 ± 4.4 mmHg at 3 h and to 20.5 ± 5.3 mmHg at 6 h after furosemide administration. Amiloride did not produce a diuretic response and was not associated with significant changes in cortical or medullary tissue oxygenation. In conclusion, clinically relevant doses of diuretic agents did not improve regional renal tissue oxygenation in healthy animals during the 8 h experimentation period. On the contrary, rebound renal cortical hypoxia may develop after dissipation of furosemide‐induced diuresis.

## Abstract (highlights)

- **What is the central question of this study?**
  What are the effects of commonly used diuretics on renal cortical and medullary perfusion and oxygenation in non‐anaesthetised healthy sheep?
- **What is the main finding and its importance?**
  Diuretic agents do not increase renal tissue oxygen tension in non‐anaesthetised healthy sheep. On the contrary, rebound cortical tissue hypoxia can develop after furosemide‐induced diuresis has dissipated.

## 1 INTRODUCTION

Diuretics are commonly prescribed for a wide range of chronic diseases including heart failure (Felker et al., 2020), hypertension (Roush et al., 2014) and chronic kidney disease (Sica, 2012). They are also commonly deployed in intensive care for management of fluid balance in critically ill patients with acute kidney injury (McCoy et al., 2019). These drugs act by inhibiting transporters situated at distinct sites along the nephron to reduce the tubular reabsorption of sodium (Kiil, 1977). Thus, they also reduce oxygen consumption within these nephron segments (Kiil, 1977). However, these actions on proximal nephron segments have the potential to increase the reabsorptive sodium load, and thus oxygen consumption, in distal nephron segments. Consequently, diuretics likely have complex and diverse actions on kidney oxygenation. This issue is of particular interest because of the implication that diuretics might either exacerbate or alleviate renal hypoxia, which has been proposed as an important driver of acute kidney injury and its transition to chronic kidney disease (Fine & Norman, 2008; Ow et al., 2018; Tanaka et al., 2014; Ullah & Basile, 2019). Technological limitations have hindered our ability to assess regional kidney oxygenation in conscious and freely moving animals (Evans et al., 2008; Ow et al., 2018). Furthermore, previous investigations of the effects of diuretics on renal oxygenation have either used indirect methods (e.g., blood oxygen level dependent magnetic resonance imaging; BOLD‐MRI) (Pedersen et al., 2007; Prasad et al., 1996; Warner et al., 2011) or large doses of diuretics beyond their clinical range (Brezis et al., 1994; Priatna et al., 1999; Warner et al., 2011). To meet this challenge, we developed an ovine model in which renal cortical and medullary tissue oxygenation and perfusion can be measured continuously in the absence of confounding effects of anaesthesia (Lankadeva et al., 2018).

The aim of the current study was to compare the effects of clinically relevant doses of three commonly used diuretics, that primarily act along distinct segments of the nephron, acetazolamide (i.e., proximal tubule), furosemide (i.e., loop of Henle) and amiloride (i.e., distal tubule) on renal medullary and cortical tissue oxygen tension ($P_{O_{2}}$) in conscious freely moving healthy sheep.

Acetazolamide is a non‐competitive and reversible inhibitor of the enzyme carbonic anhydrase which is chiefly localised to the basolateral membrane of the S2 and S3 segments of the proximal tubules and in the collecting ducts (Purkerson & Schwartz, 2007). Carbonic anhydrase facilitates the transepithelial reabsorption of bicarbonate coupled to the secretion of protons and subsequently reabsorption of sodium via Na^+^/H^+^ exchanger 3 (NHE3) transporters (Krishnan et al., 2015). We hypothesised that acetazolamide increases cortical tissue oxygenation but promotes renal medullary tissue hypoxia. Furosemide inhibits the Na^+^–K^+^–Cl^−^ cotransporter on the luminal side of the thick ascending limb of the cortex and medulla (Castrop & Schießl, 2014; Huang et al., 2016; Orlov et al., 2015). We hypothesised that furosemide increases renal medullary tissue oxygen $P_{O_{2}}$. Amiloride is a reversible inhibitor of the epithelial sodium channels in the luminal membrane of the collecting ducts (Mutchler & Kleyman, 2019; Sun & Sever, 2020). Since amiloride acts chiefly on the distal portion of the nephrons, we hypothesised that amiloride does not alter renal cortical $P_{O_{2}}$.

## 2 METHODS

### 2.1 Ethical approval

All experimental procedures were approved by the Animal Ethics Committee of the Florey Institute of Neuroscience and Mental Health (Approval number: 17‐009‐FINMH and 19‐049‐FINMH) under the guidelines of the National Health and Medical Research Council of Australia and conformed with the ARRIVE and ARRIVE 2.0 guidelines (Percie du Sert et al., 2020). Merino ewes (1.5–2.0 years) were housed in pens for acclimatisation at least a week prior to experimentation. Thereafter, they were transferred and housed individually in metabolic cages for the duration of the experiment. Ewes were allowed free access to oaten chaff and water.

### 2.2 Surgical preparation

Each sheep underwent two preparative surgical procedures under general anaesthesia. Induction of anaesthesia was achieved with intravenous sodium thiopentone (15 mg/kg, Jurox Pty Ltd, Rutherford, NSW, Australia) and maintained with isoflurane (2.0–2.5% v/v oxygen/air/isoflurane) once the sheep was intubated. Prior to the first incision, the sheep was given 900 mg of the antibiotic procaine penicillin (Ilium Propercillin, Troy Laboratories, Glendenning, NSW, Australia) and 1 mg/kg of the analgesic flunixin meglumine (Ilium Flunixil, Troy Laboratories) intramuscularly. Post‐surgical analgesic was also administered 4 and 24 h after surgery while additional antibiotic was administered 24 and 48 h after surgery.

As described in detail previously (Lankadeva et al., 2018), during the first surgical procedure, a carotid arterial loop was constructed, so that the left carotid artery was exteriorised into a skin fold, to facilitate arterial cannulation for sampling of blood and measurement of arterial pressure. A transit‐time flow probe (20 mm, Transonic Systems Inc., Ithaca, NY, USA) was then implanted around the pulmonary artery for measurement of cardiac output.

After a 2–3 week recovery period, the left carotid artery and jugular vein were cannulated to facilitate arterial blood sampling and intravenous infusion of drugs. The sheep underwent abdominal surgery, as described in detail previously (Lankadeva et al., 2018), during which the left renal vein and artery and the left kidney were exposed. The renal vein was cannulated, and the catheter exteriorised so that renal venous blood could be sampled. A transit‐time flow probe (4 mm, Transonic Systems Inc.) was placed around the left renal artery for measurement of renal blood flow (RBF). The left kidney was also instrumented with two custom‐built combination fibre‐optic probes (450 μm outer diameter, CP‐0004‐0001, Oxford Optronix, Adderbury, UK). Each probe comprised a dual‐fibre laser Doppler probe for assessment of tissue perfusion, a fluorescence optode for measurement of tissue $P_{O_{2}}$ and a thermistor for measurement of tissue temperature. The position of the probes was confirmed at necropsy. A Foley catheter (14 Fr, Livingstone International Pty Ltd, Mascot, NSW, Australia) was advanced into the bladder to enable later sampling of urine and measurement of urinary $P_{O_{2}}$ (section 2.3).

### 2.3 Experimental protocol and calculations

After 3–5 days of recovery from the second surgical procedure, an intravenous maintenance infusion of 0.9% v/v sodium chloride commenced at 1 mL/kg/h in conscious sheep overnight during baseline recording. We have previously measured plasma levels of pro‐ and anti‐inflammatory cytokines, total white blood cell counts, monocytes and neutrophils from sheep 3 days after recovery from surgery (Lankadeva et al., 2019, 2020; Peiris et al., 2023) and established that they were within the levels observed in plasma from naïve sheep (Bouquet et al., 2020; Neeland et al., 2014). The infusion continued for the remainder of the experimental period. To permit measurement of bladder urinary $P_{O_{2}}$, a large area sensor oxygen (LAS‐1/O/E, Oxford Optronix) probe was inserted into a port connected to the Foley bladder catheter and advanced to the tip of the catheter within the bladder. After an hour of baseline recording, sheep received either acetazolamide (*n* = 9, body weight = 40.7 ± 1.7 kg, 250 mg, A6011, Sigma‐Aldrich Pty Ltd, Bayswater, VIC, Australia), furosemide (*n* = 10, body weight = 38.6 ± 1.4 kg, 20 mg, Lasix®, Sanofi‐Aventis Australia Pty Ltd, Macquarie Park, NSW, Australia) or amiloride (*n* = 7, body weight = 39.6 ± 1.3 kg, 10 mg, A7410, Sigma‐Aldrich Pty Ltd, Bayswater, VIC, Australia) as an intravenous bolus over 30 min. At the end of the experiment, the sheep were killed with sodium pentobarbitone administered intravenously (20 mg/kg, Lethobarb, Virbac, Wetherill Park, Australia).

Arterial and renal venous blood were sampled, before and then at 4 and 8 h after diuretic treatment, for blood oximetry (ABL system 625, Radiometer Medical, Copenhagen, Denmark) and for assessment of arterial plasma creatinine concentration. Hourly urinary output was recorded. Urine samples were collected over 1 h periods ending just before the blood samples were collected, before and at the fourth and eighth hour after treatment commenced. Plasma and urinary concentrations of creatinine and sodium were then measured so that renal excretory functions could be assessed. Mean arterial pressure (MAP), heart rate (HR, triggered by the arterial pulse pressure), RBF, cardiac output, renal cortical and medullary tissue perfusion, tissue $P_{O_{2}}$ and temperature, and bladder urinary $P_{O_{2}}$ were continuously recorded for the duration of the experiment. Renal oxygen delivery ($RD_{O_{2}}$) was calculated as the product of the arterial blood concentration of oxygen and RBF, while renal oxygen consumption ($R{\dot{V}}_{O_{2}}$) was calculated as the product of the arteriovenous oxygen concentration difference and RBF. Fractional renal oxygen extraction was calculated as $R{\dot{V}}_{O_{2}}$ expressed as a percentage of $RD_{O_{2}}$. Plasma and urinary concentrations of creatinine and the urinary concentration of sodium were measured at the Pathology Service at Austin Health, Melbourne, VIC, Australia. Total peripheral conductance (TPC) was calculated as cardiac output/MAP and renal vascular conductance (RVC) was calculated as RBF/MAP.

### 2.4 Statistical analysis

The plasma half‐lives of acetazolamide (4–8 h) (Van Berkel & Elefritz, 2018), furosemide (30 min to 2 h) (Van Wart et al., 2014) and amiloride (6–9 h) (Sun & Sever, 2020) differ. Thus, some variation in the time course of their diuretic effects in sheep was expected. Therefore, in our analysis of these data, we first assessed the time course of their diuretic effects so that analysis of the effects on renal oxygenation could be linked to the respective windows of maximum diuresis of each agent. Statistical analyses and figures were generated using the software GraphPad Prism (Version 9.0, GraphPad Software, Boston, MA, USA). Data are expressed as means ± SD. Two‐sided *P* ≤ 0.05 was considered statistically significant. One‐way repeated measures analysis of variance (ANOVA) was used to assess the effects of drug treatment on parameters assessed continuously over time. *P*‐values derived from these within‐subjects factors in repeated‐measures ANOVA were conservatively adjusted using the Greenhouse–Geisser method (Ludbrook, 1994). Post‐hoc multiple comparisons to compare time points after diuretic administration with baseline were conducted using Dunnett's test.

## 3 RESULTS

**FIGURE 1:**
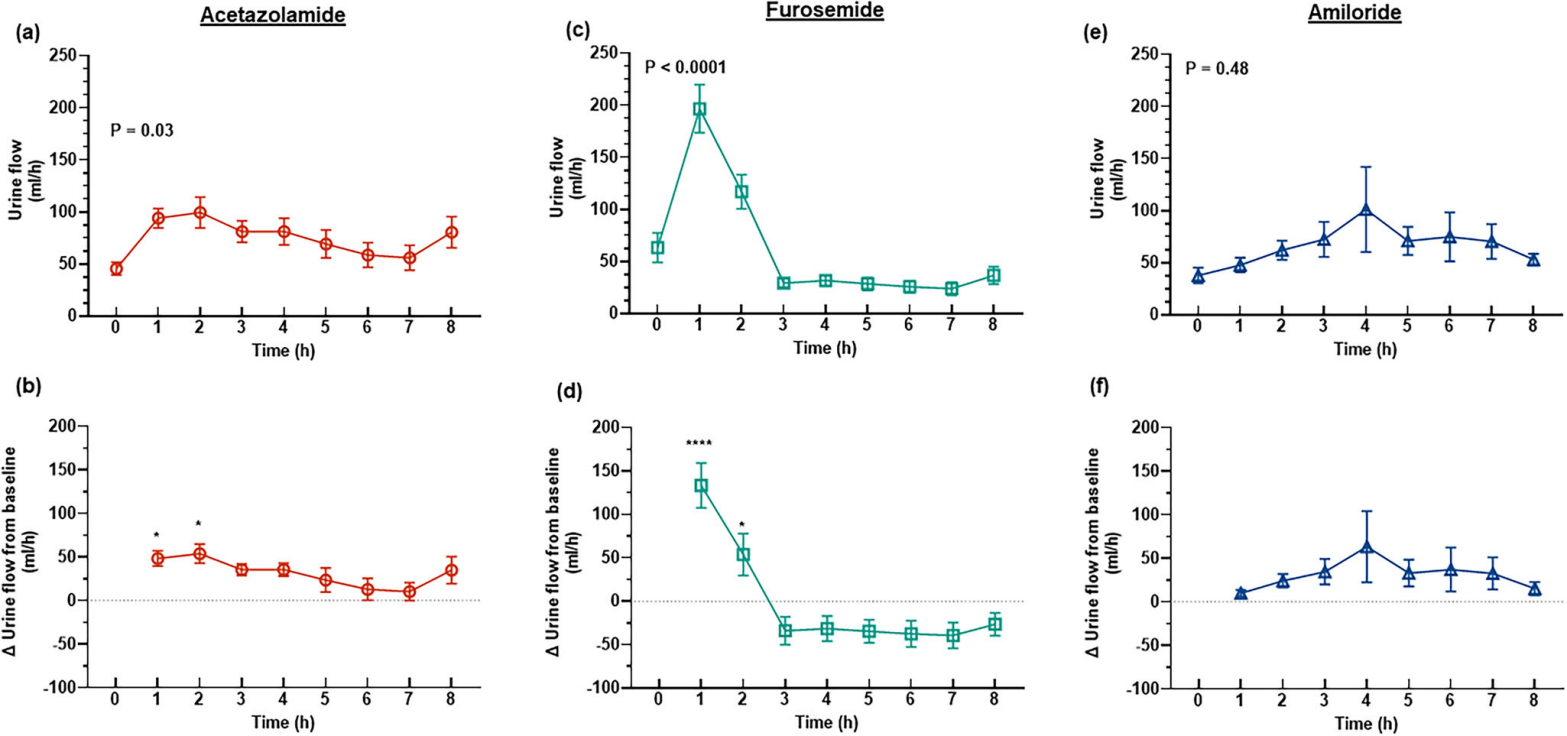
Urine flow. Hourly urine output (a, c, e) and the change in urine flow from baseline (b, d, f) after administration of 250 mg acetazolamide (a–b, *n* = 9), 20 mg furosemide (c–d, *n* = 10) or 10 mg amiloride (e–f, *n* = 7). Values are presented as means ± SD. In (a, c, e) *P*‐values are the outcomes of one‐way ANOVA over the 8 h experimental period for each drug treatment. In (b, d, f) asterisks denote post‐hoc Dunnett's multiple comparisons to the baseline of each group. *P ≤ 0.05, ****P ≤ 0.0001.

**FIGURE 2:**
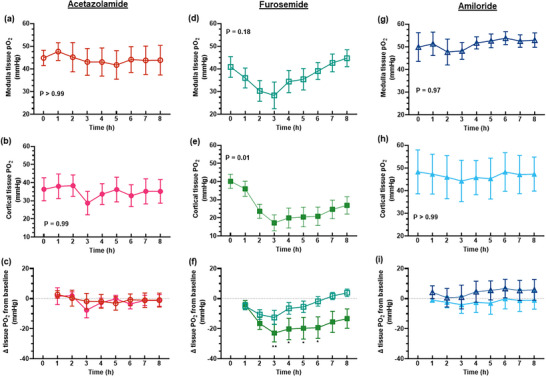
Regional kidney tissue oxygen tension. Values are presented as means ± SD for medullary (a, d, g) and cortical (b, e, h) tissue oxygen tension and the change in regional kidney tissue oxygen tension from baseline (c, f, i) after administration of 250 mg acetazolamide (a–c, *n* = 9), 20 mg furosemide (d–f, *n* = 10) or 10 mg amiloride (g–i, *n* = 7). In (a, b, d, e, g, h) *P*‐values are the outcomes of one‐way ANOVA over the 8 h experimental period for each drug treatment. In (c, f, i) asterisks denote post‐hoc Dunnett's multiple comparisons to the baseline of each group. *P ≤ 0.05, **P ≤ 0.01. Open symbols denote medullary tissue $P_{O_{2}}$_,_ and filled symbols denote cortical tissue $P_{O_{2}}$.

**FIGURE 3:**
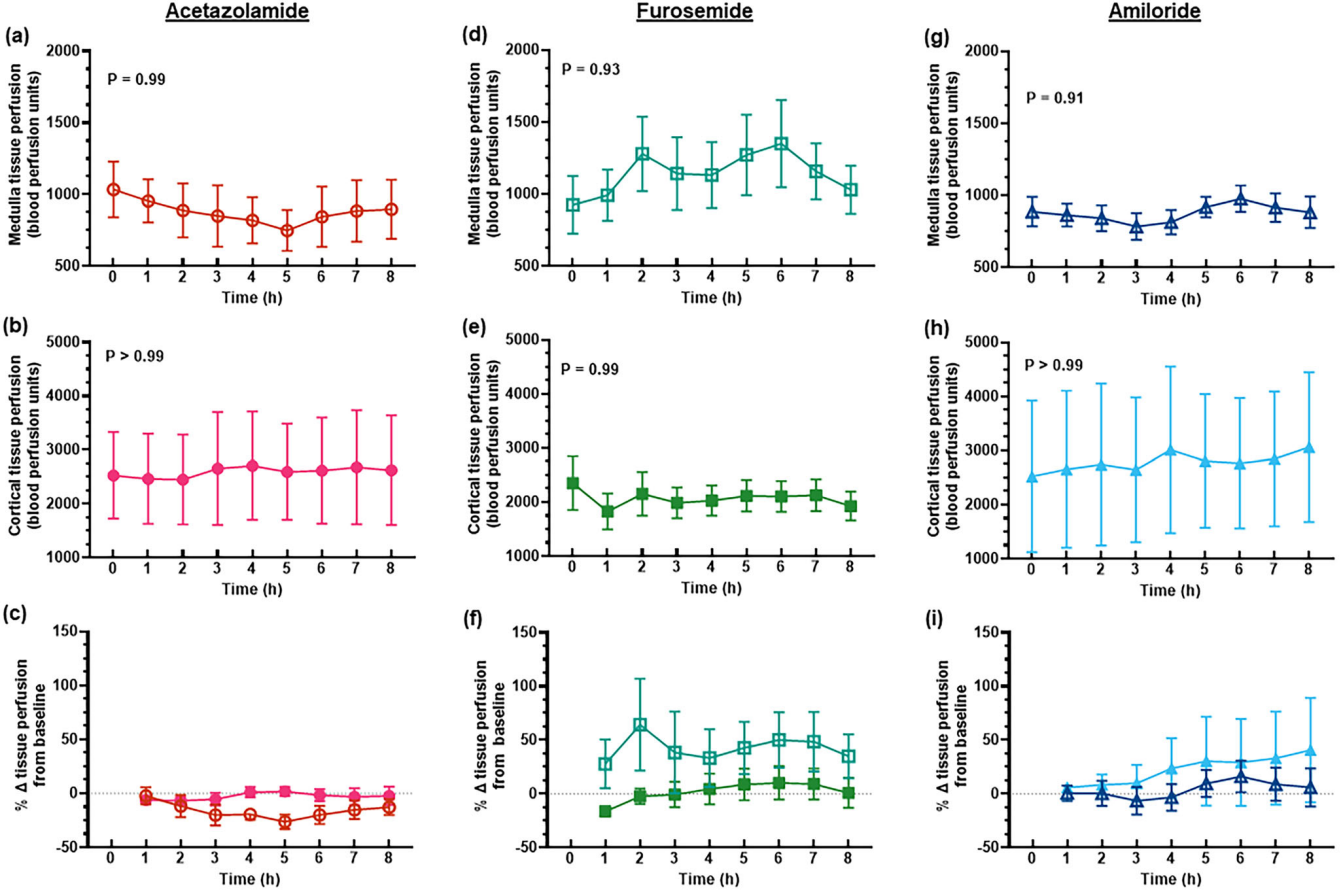
Regional kidney tissue perfusion. Values are presented as means ± SD for medullary (a, d, g) and cortical (b, e, h) tissue perfusion and the change in regional kidney tissue perfusion from baseline (c, f, i) after administration of 250 mg acetazolamide (a–c, *n* = 9), 20 mg furosemide (d–f, *n* = 10) or 10 mg amiloride (g–i, *n* = 7). In (a, b, d, e, g, h) *P*‐values are the outcomes of one‐way ANOVA over the 8 h experimental period for each drug treatment. Open symbols denote medullary tissue perfusion and filled symbols denote cortical tissue perfusion.

**FIGURE 4:**
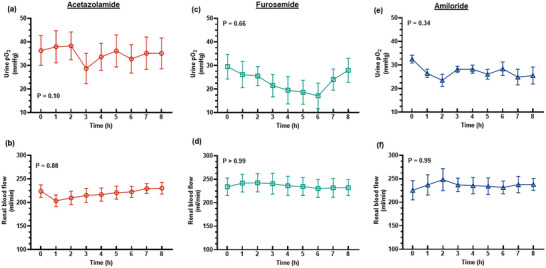
Urinary oxygen tension and total renal blood flow. Values are presented as means ± SD for urinary oxygen tension (a, c, e) and renal blood flow (b, d, f) after administration of 250 mg acetazolamide (*n* = 9), 20 mg furosemide (*n* = 10) or 10 mg amiloride (*n* = 7). *P*‐values are the outcomes of one‐way ANOVA over the 8 h experimental period for each drug treatment.

**FIGURE 5:**
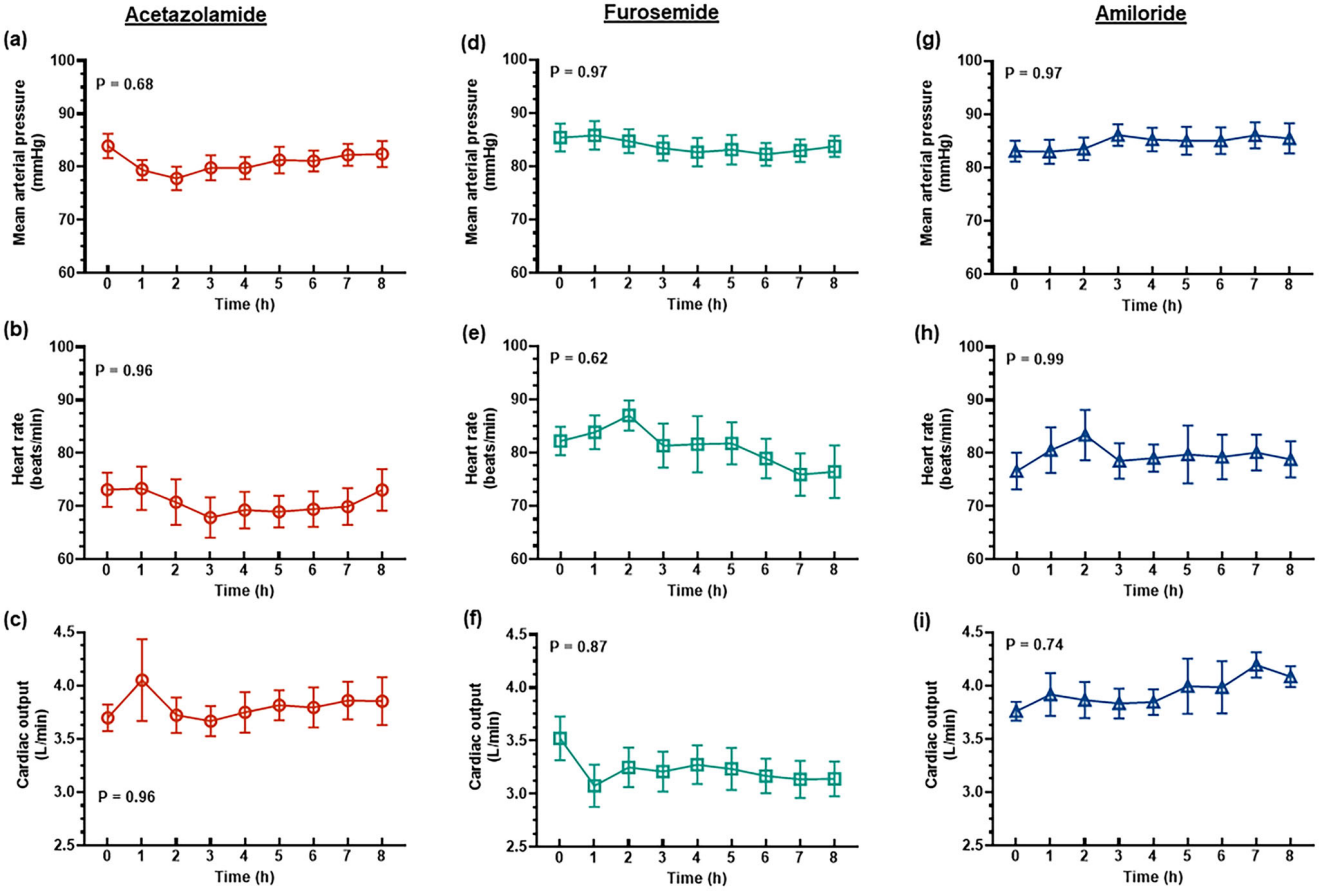
Systemic haemodynamic variables. Values are presented as means ± SD for mean arterial pressure (a, d, g), heart rate (b, e, h) and cardiac output (c, f, i) after bolus administration of 250 mg acetazolamide, 20 mg furosemide or 10 mg amiloride. *P*‐values are the outcomes of one‐way ANOVA over the 8 h experimental period for each drug treatment. (Mean arterial pressure and heart rate: acetazolamide: *n* = 9, furosemide: *n* = 10, amiloride: *n* = 7; cardiac output: acetazolamide: *n* = 6, furosemide: *n* = 9, amiloride: *n* = 4.)

**FIGURE 6:**
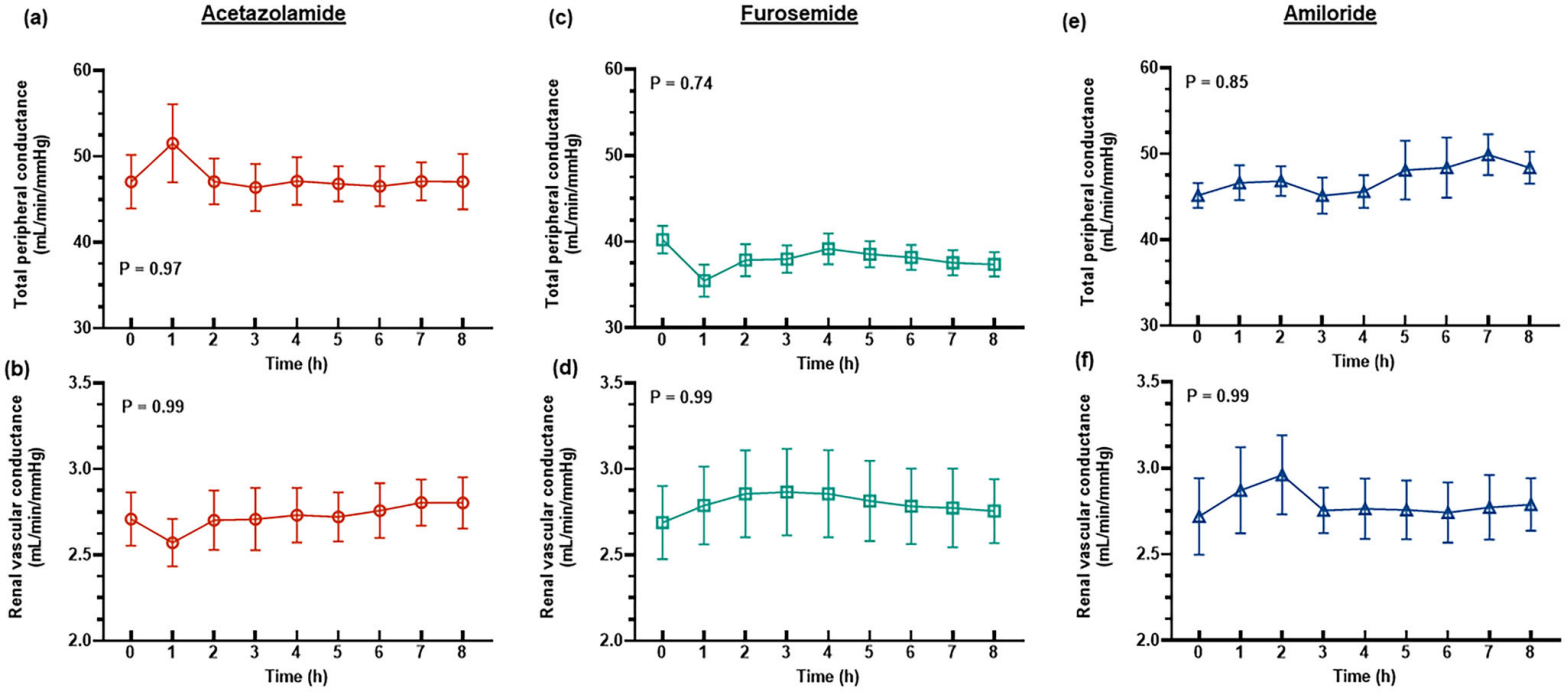
Systemic and renal haemodynamic parameters. Values are presented as means ± SD for total peripheral conductance (a, c, e) and renal vascular conductance (b, d, f) after bolus administration of 250 mg acetazolamide, 20 mg furosemide or 10 mg amiloride. *P*‐values are the outcomes of one‐way ANOVA over the 8 h experimental period for each drug treatment. (Total peripheral conductance: acetazolamide: *n* = 6, furosemide: *n* = 9, amiloride: *n* = 4; renal vascular conductance: acetazolamide: *n* = 9, furosemide: *n* = 9, amiloride: *n* = 7.)

The diuretic response to acetazolamide was apparent for approximately the first 4 h after administration of the bolus, although the increase in urine flow from baseline was only statistically significant for the first 2 h (Figure 1a). The peak increase in urine flow from baseline occurred at 2 h post‐treatment from 45.6 ± 18.5 to 99.4 ± 44.4 mL/h (127.1 ± 75.6%, *P* = 0.015, Figure 1b). During the period of diuresis, there were no significant changes in renal cortical or medullary $P_{O_{2}}$ (Figure 2a–c) or tissue perfusion (Figure 3a–c). Similarly, neither total RBF nor urinary $P_{O_{2}}$ changed significantly after acetazolamide (Figure 4a,b). Furthermore, there were no significant changes in the major determinants of renal oxygenation, $RD_{O_{2}}$ and $R{\dot{V}}_{O_{2}}$ (Table 1). Consequently, renal fractional extraction of oxygen was not significantly different following treatment (Table 1). There were also no apparent changes in systemic haemodynamics (Figures 5a–c and 6a,b). The blood concentration of bicarbonate was significantly decreased at 4 h (−11.3 ± 8.5%, *P* = 0.008) and 8 h (−14.2 ± 12.2%, *P* = 0.001) after administration of acetazolamide (Table 2). Similarly, arterial blood lactate concentration was significantly less than the baseline at 4 h (−36.1 ± 30.4%, *P* = 0.002) and 8 h (−36.0 ± 17.4%, *P* = 0.004) after treatment (Table 2). Consequently, these changes resulted in the arterial blood pH being significantly lower over time. There was no significant change in creatinine clearance (Table 3). However, there was a significant increase in urinary sodium excretion (*P* = 0.004, Table 3) and fractional excretion of sodium at 4 h post‐treatment (*P* = 0.005, Table 3).

**TABLE 1:** Renal oxygen delivery and consumption.

Parameter	Acetazolamide				Furosemide				Amiloride			
	Baseline	4 h	8 h	One‐way ANOVA	Baseline	4 h	8 h	One‐way ANOVA	Baseline	4 h	8 h	One‐way ANOVA
Arterial blood oxygen content (mL O_2_/dL)	13.1 ± 2.0 (9)	12.9 ± 1.8 (9)	13.1 ± 1.6 (9)	0.965	12.0 ± 1.3 (10)	12.1 ± 1.5 (10)	11.8 ± 1.4 (10)	0.920	12.3 ± 1.7 (7)	12.5 ± 1.7 (7)	11.6 ± 1.6 (7)	0.612
Renal venous blood oxygen content (mL O_2_/dL)	11.6 ± 1.7 (9)	11.3 ± 2.1 (9)	11.1 ± 1.1 (9)	0.849	10.4 ± 1.7 (9)	11.0 ± 1.7 (9)	10.3 ± 1.6 (8)	0.737	10.9 ± 1.5 (7)	11.3 ± 1.3 (7)	10.6 ± 1.4 (7)	0.645
Renal oxygen delivery (mL O_2_/dL)	29.6 ± 7.7 (9)	28.1 ± 6.9 (9)	30.4 ± 6.8 (9)	0.792	27.8 ± 7.0 (9)	27.8 ± 7.3 (9)	26.7 ± 5.2 (9)	0.917	28.1 ± 9.2 (7)	29.4 ± 6.8 (7)	27.8 ± 5.9 (7)	0.919
Renal oxygen consumption (mL O_2_/dL)	3.0 ± 1.9 (8)	2.9 ± 1.7 (8)	2.9 ± 1.9 (8)	0.219	3.3 ± 1.9 (7)	2.6 ± 1.1 (7)	2.6 ± 1.2 (7)	0.595	3.5 ± 3.3 (7)	2.9 ± 2.2 (7)	2.5 ± 1.7 (7)	0.761
Fractional extraction of oxygen (%)	10.6 ± 5.0 (8)	11.6 ± 7.0 (8)	14.4 ± 3.5 (8)	0.346	13.1 ± 6.1 (7)	10.4 ± 4.8 (7)	10.6 ± 5.5 (7)	0.611	10.9 ± 7.1 (7)	9.0 ± 6.0 (7)	8.7 ± 5.9 (7)	0.772

Values are means ± SD (*n*) for the baseline period and at the 4th and 8th hour after sheep were treated with acetazolamide (250 mg), furosemide (20 mg) or amiloride (10 mg). *P*‐values are outcomes of one‐way repeated measures ANOVA factor.

**TABLE 2:** Arterial blood oximetry.

Parameter	Acetazolamide				Furosemide				Amiloride			
	Baseline	4 h	8 h	One‐way ANOVA	Baseline	4 h	8 h	One‐way ANOVA	Baseline	4 h	8 h	One‐way ANOVA
pH	7.49 ± 0.04 (9)	7.46 ± 0.04 (9)	7.44 ± 0.03** (9)	0.014	7.51 ± 0.04 (10)	7.51 ± 0.03 (10)	7.51 ± 0.04 (10)	0.993	7.51 ± 0.02 (7)	7.59 ± 0.03 (7)	7.48 ± 0.07 (7)	0.763
$P_{CO_{2}}$ (mmHg)	32.7 ± 3.1 (9)	32.0 ± 3.6 (9)	31.7 ± 3.6 (9)	0.818	32.2 ± 4.6 (10)	33.8 ± 1.4 (9)	34.0 ± 1.7 (9)	0.354	33.7 ± 2.2 (7)	33.3 ± 3.1 (7)	32.4 ± 4.4 (7)	0.759
$P_{O_{2}}$ (mmHg)	101.4 ± 8.5 (9)	105.3 ± 11.8 (9)	109.3 ± 10.4 (9)	0.288	92.6 ± 5.6 (10)	93.0 ± 4.5 (10)	95.7 ± 5.9 (10)	0.379	97.2 ± 11.6 (7)	102.0 ± 14.3 (7)	109.3 ± 10.5 (7)	0.203
Haemoglobin (g/L)	9.5 ± 1.4 (9)	9.4 ± 1.2 (9)	9.5 ± 1.1 (9)	0.960	8.7 ± 1.0 (10)	8.8 ± 0.3 (10)	8.6 ± 0.3 (10)	0.905	9.0 ± 0.5 (7)	9.1 ± 0.5 (7)	8.4 ± 0.4 (7)	0.486
$S_{O_{2}}$ (%)	96.7 ± 1.3 (9)	96.7 ± 1.7 (9)	96.7 ± 1.8 (9)	0.998	96.6 ± 1.5 (10)	96.8 ± 1.5 (10)	96.8 ± 1.5 (10)	0.959	96.1 ± 2.2 (7)	96.2 ± 2.2 (7)	97.0 ± 1.0 (7)	0.632
Potassium (mmol/L)	4.01 ± 0.50 (9)	3.97 ± 0.42 (9)	4.23 **± **0.47 (9)	0.444	4.21 ± 0.60 (10)	4.08 ± 0.86 (10)	4.17 ± 0.73 (10)	0.922	3.96 ± 0.45 (7)	4.17 ± 0.37 (7)	4.07 ± 0.44 (7)	0.381
Sodium (mmol/L)	140.3 ± 2.7 (9)	139.3 ± 3.2 (9)	139.3 ± 3.9 (9)	0.765	140.3 ± 7.3 (10)	143.2 ± 2.4 (10)	143.2 ± 2.6 (10)	0.308	139.7 ± 2.1 (7)	138.4 ± 3.8 (7)	140.0 ± 4.6 (7)	0.699
Ionized calcium (mmol/L)	1.13 ± 0.10 (9)	1.16 ± 0.07 (9)	1.19 ± 0.04 (9)	0.285	1.20 ± 0.07 (10)	1.22 ± 0.06 (10)	1.24 ± 0.07 (10)	0.398	1.13 ± 0.09 (7)	1.14 ± 0.05 (7)	1.13 ± 0.11 (7)	0.910
Chloride (mmol/L)	108.6 ± 3.5 (9)	110.4 ± 3.7 (9)	110.8 ± 3.3 (9)	0.364	107.9 ± 1.7 (10)	107.7 ± 1.5 (10)	108.5 ± 1.4 (10)	0.0849	106.4 ± 2.0 (7)	106.9 ± 4.5 (7)	108.7 ± 4.6 (7)	0.515
HCO_3_^−^ (mmol/L)	24.9 ± 2.1 (9)	22.0 ± 1.9** (9)	21.2 ± 1.8*** (9)	0.001	25.4 ± 3.1 (10)	26.8 ± 2.3 (9)	26.6 ± 1.8 (9)	0.417	26.1 ± 1.5 (7)	24.7 ± 3.1 (7)	24.0 ± 3.0 (7)	0.316
Lactate (mmol/L)	0.58 ± 0.13 (9)	0.36 ± 0.14** (9)	0.37 ± 0.11** (9)	0.002	0.53 ± 0.27 (8)	0.35 ± 0.06 (8)	0.38 ± 0.20 (8)	0.181	0.50 ± 0.16 (7)	0.43 ± 0.10 (7)	0.041 ± 0.11 (7)	0.409

Values are means ± SD (*n*) for arterial blood drawn for oximetry at the end of the baseline period and at the 4th and 8th hours after sheep were treated with acetazolamide (250 mg), furosemide (20 mg) or amiloride (10 mg). *P*‐values are outcomes of one‐way repeated measures ANOVA. Asterisks denote post‐hoc Dunnett's multiple comparisons to the baseline of each group. **P ≤ 0.01, ***P ≤ 0.001.

**TABLE 3:** Indices of renal excretory function.

Parameter	Acetazolamide				Furosemide				Amiloride			
	Baseline	4 h	8 h	One‐way ANOVA	Baseline	4 h	8 h	One‐way ANOVA	Baseline	4 h	8 h	One‐way ANOVA
Na^+^ excretion (mmol/min)	0.09 ± 0.02 (8)	0.24 ± 0.04** (8)	0.18 ± 0.03 (8)	0.008	0.05 ± 0.01 (10)	0.04 ± 0.01 (10)	0.04 ± 0.01 (10)	0.727	0.10 ± 0.02 (6)	0.20 ± 0.04* (6)	0.15 ± 0.01 (6)	0.040
Fractional excretion of Na^+^ (%)	0.90 ± 0.46 (8)	2.26 ± 1.0** (8)	1.65 ± 0.78 (8)	0.009	2.73 ± 6.07 (10)	2.89 ± 6.90 (10)	2.76 ± 7.02 (10)	0.998	0.81 ± 0.40 (6)	1.73 ± 1.18 (6)	1.30 ± 0.60 (6)	0.168
Plasma creatinine (μmol/L)	65.7 ± 6.8 (9)	66.7 ± 7.1 (9)	64.9 ± 7.1 (9)	0.866	151.7 ± 141.1 (10)	155.0 ± 152.3 (10)	150.8 ± 154.4 (10)	0.998	59.8 ± 6.8 (6)	57.8 ± 8.2 (6)	56.2 ± 8.1 (6)	0.851
Creatinine clearance (mL/min)	69.5 ± 28.1 (9)	73.5 ± 16.9 (9)	79.2 ± 32.5 (9)	0.742	44.9 ± 30.7 (10)	39.7 ± 23.2 (10)	51.9 ± 31.3 (10)	0.639	81.3 ± 16.3 (6)	88.3 ± 23.7 (6)	86.5 ± 23.8 (6)	0.847

Values are means ± SD (*n*) for the baseline period and at the end of the 4th and 8th hour after sheep were treated with acetazolamide (250 mg), furosemide (20 mg) or amiloride (10 mg). *P*‐values are outcomes of one‐way repeated measures ANOVA. Asterisks denote post‐hoc Dunnett's multiple comparisons to the baseline of each group. **P ≤ 0.01.

The diuretic response to furosemide occurred rapidly (Figure 1c,d). The peak diuretic response occurred 1 h after treatment (*P* < 0.0001). Thereafter, the urine output steadily declined such that by 3 h after treatment, urine flow was no longer statistically distinguishable from its baseline level. Neither medullary tissue $P_{O_{2}}$ nor perfusion changed significantly after furosemide during the period of diuresis (Figures 2d,f and 3d,f). In contrast, relative cortical hypoxia developed after cessation of the diuretic response of furosemide, such that cortical tissue $P_{O_{2}}$ was between 19.7 ± 22.9 and 22.9 ± 18.6 mmHg less than its baseline level (40.1 ± 12.2 mmHg) from 3 to 6 h after treatment (Figure 2e,f). Interestingly, this effect occurred in the absence of significant changes in cortical perfusion (Figure 3e,f). There was no significant change in total RBF after furosemide treatment (Figure 4a). There was a tendency for bladder urinary $P_{O_{2}}$ to decrease once the diuresis has dissipated in furosemide‐treated sheep, but this apparent effect did not reach statistical significance (Figure 4b). Furthermore, there were no significant changes in $RD_{O_{2}}$, $R{\dot{V}}_{O_{2}}$ or the fractional extraction of oxygen (Table 1). As was the case to acetazolamide, furosemide treatment in healthy sheep was not followed by any apparent changes in systemic haemodynamics (Figures 5d–f and 6c,d). There were also no significant changes in arterial blood chemistry (Table 2) or renal excretory function (Table 3).

The diuretic response to amiloride was modest in that the peak urine flow that occurred at 4 h post‐treatment was statistically indistinguishable from the baseline (Figure 1e,f). There were no apparent changes in renal medullary or cortical tissue $P_{O_{2}}$ (Figure 2g–i) or perfusion (Figure 3g–i), urinary $P_{O_{2}}$ or total RBF (Figure 4e,f). Likewise, there were no significant changes in systemic haemodynamics (Figure 5g–i and e,f), $RD_{O_{2}}$, $R{\dot{V}}_{O_{2}}$ or fractional extraction of oxygen in the kidney (Table 1), arterial blood chemistry (Table 2) or renal excretory function (Table 3) in response to amiloride. There was, however, a moderate increase in urinary excretion of sodium at 4 h after treatment (*P* = 0.023, Table 3) but this natriuretic effect was no longer statistically significant at 8 h after treatment.

## 4 DISCUSSION

We found that acetazolamide and amiloride, when administered at therapeutic doses, to non‐anaesthetised but otherwise healthy sheep, did not alter renal cortical or medullary tissue $P_{O_{2}}$. However, we did find that selective hypoxia developed in the renal cortex, after the diuretic response to furosemide had dissipated. Thus, our findings contradict the proposition that clinically relevant doses of these diuretic agents increase renal tissue $P_{O_{2}}$, at least in healthy sheep. Our findings also raise the prospect that rebound tissue hypoxia, after the diuretic effect of furosemide has waned, might reduce renal cortical tissue $P_{O_{2}}$.

Diuretics are commonly prescribed therapeutics for both acute and chronic conditions in patients with or without renal co‐morbidities. A proposed potential therapeutic benefit of diuretics is to alleviate regional renal hypoxia through reduction in oxygen consumed for sodium reabsorption (Kiil, 1977). One of the impediments to our understanding of the regulation and dysregulation of regional renal tissue oxygenation has been the lack of methods for direct assessment of renal tissue oxygenation without the confounding effects of anaesthesia (Evans et al., 2008; Ow et al., 2018). In the current study, using a method we developed for direct assessment of regional kidney tissue $P_{O_{2}}$ in conscious sheep (Lankadeva et al., 2018), we were able to assess the effects of therapeutic doses of acetazolamide, furosemide and amiloride on renal macro‐ and micro‐circulatory perfusion and oxygenation in non‐anaesthetised sheep.

Acetazolamide, acting predominantly on the proximal tubule, indirectly reduces sodium reabsorption and consequently has been proposed to improve cortical tissue oxygenation (Brezis et al., 1994). Warner and colleagues showed increased cortical and medullary oxygenation during acetazolamide‐induced diuresis using BOLD‐MRI, a non‐invasive but indirect method for assessment of renal oxygenation (Warner et al., 2011). In contrast, other studies using BOLD‐MRI, failed to show any changes in cortical and medullary tissue oxygenation in healthy rodents (Priatna et al., 1999) or humans (Prasad et al., 1996). On the other hand, Brezis and colleagues, using direct but invasive Clark‐type electrodes for assessment of tissue oxygenation, showed acetazolamide increased renal cortical tissue $P_{O_{2}}$ in anaesthetised healthy rats (Brezis et al., 1994). The dose of acetazolamide used in studies of renal tissue oxygenation conducted in experimental animals was 15 mg/kg (Warner et al., 2011) and 100 mg/kg (Brezis et al., 1994; Priatna et al., 1999), approximately 60–400 times that we used in the current study. The use of supratherapeutic doses of acetazolamide is likely to result in off‐target effects, such as inhibition and ubiquitination of aquaporin‐1 channels (Zhang et al., 2012). The loss of aquaporin‐1 has been shown to contribute to defects in urinary concentrating ability and may potentially contribute to significant changes in renal tissue oxygenation (Abdeen et al., 2016). Thus, the effects of these large doses on renal oxygenation should be interpreted with caution in consideration of their limited applicability to the clinical setting. Accordingly, in the current study, we used dosages of acetazolamide, furosemide and amiloride that are commonly used in the clinic. Simulations derived from a mathematical model developed by Layton and colleagues indicate that complete inhibition of the NHE3 antiporter reduces Na^+^/K^+^‐ATPase activity by ∼40% with an associated reduction in sodium reabsorption (Layton et al., 2015). Furthermore, their simulations indicated that inhibition of NHE3 is unlikely to greatly increase distal delivery of sodium to nephron segments. These distal tubules of the nephron are purported to be less oxygen efficient for reabsorption of sodium than the proximal tubule (Layton et al., 2016). Accordingly, computational models of the effects of acetazolamide on renal oxygenation indicate that it is unlikely to significantly alter cortical and medullary tissue oxygenation under physiological conditions. Our in vivo physiological studies in non‐anaesthetised sheep support the proposition that acetazolamide, at clinically relevant doses, is unlikely to either improve or compromise regional kidney tissue oxygenation. On the other hand, there is good evidence that the absence of NHE3 transporters in a knock‐out mouse model is associated with increased distal delivery of bicarbonate and with it sodium (Bailey et al., 2004). The increased sodium load at the less metabolically efficient distal nephrons could potentially lead to increased renal oxygen consumption and thus potentially reduced renal tissue $P_{O_{2}}$. Consistent with this proposition, in the current experiments, acetazolamide increased both total sodium excretion and the fractional excretion of sodium, indicating increased delivery of sodium to the distal nephron. However, this effect was not associated with significant changes in renal tissue $P_{O_{2}}$.

Brezis and colleagues reported that inhibition of sodium transport in the highly metabolically active medullary thick ascending limb of the loop of Henle using furosemide increased medullary tissue $P_{O_{2}}$ by approximately two‐fold while the cortical $P_{O_{2}}$ was relatively unaffected (Brezis et al., 1994). The caveat to this observation is that the dose of furosemide used was 10 mg/kg, approximately 20 times the dose we used in the current study and the study was conducted in anaesthetised animals. Using BOLD‐MRI, there have been consistent observations of improved medullary and/or cortical tissue oxygenation after similarly high doses of furosemide in experimental animals (Lee et al., 2021; Pedersen et al., 2007; Priatna et al., 1999). Interestingly, using BOLD‐MRI, improvements in renal oxygenation were also observed in healthy human volunteers given clinically relevant doses of furosemide (Epstein & Prasad, 2000; Haddock et al., 2019; Lal et al., 2022; Prasad et al., 1996). However, BOLD‐MRI is an indirect method for assessment of renal tissue oxygenation as it measures the ratio of oxyhaemoglobin to deoxyhaemoglobin (Evans et al., 2008; Ow et al., 2018). Thus, it provides an index of blood oxygenation and not tissue $P_{O_{2}}$ (Evans et al., 2008; Ow et al., 2018). Furthermore, it is heavily influenced by water content as the principle of BOLD‐MRI is based on the electro‐paramagnetism of the proton signal of water molecules in blood (Evans et al., 2008). In contrast, using a direct method, we observed little acute effect of furosemide on renal tissue $P_{O_{2}}$ during the period of diuresis. However, relative renal cortical hypoxia developed during the period after furosemide‐induced diuresis had waned. These observations, while strikingly dissimilar to those of previous studies of renal oxygenation (Brezis et al., 1994; Epstein & Prasad, 2000; Priatna et al., 1999), accord with simulations derived from the mathematical model developed by Layton and colleagues. According to their simulations, inhibition of the Na^+^–K^+^–Cl^−^ cotransporter is predicted to be associated with a marked increase in delivery of sodium to distal parts of the nephron (i.e., distal convoluted tubule, cortical collecting ducts and connecting tubules in the cortex) (Layton et al., 2016). Furthermore, at 100% inhibition, the efficiency of oxygen utilisation for sodium reabsorption at the whole kidney level is predicted to decrease (Layton et al., 2016), potentially exposing the renal cortex to increased propensity for development of tissue hypoxia, which aligns with our current findings. We can only speculate regarding the mechanisms underlying the relative cortical hypoxia which developed during the period 3–6 h after administration of furosemide. One possible contributing factor could be increased distal delivery of sodium, and hence reabsorption of sodium by distal nephron segments, as outlined above. However, our ability to temporally resolve changes in renal sodium handling is limited by the fact that urinary sodium excretion was only measured at baseline and during the fourth and eighth hour after administration of furosemide. It is also noteworthy that mean plasma creatinine in the cohort of sheep treated with furosemide was numerically greater but not significantly greater than that of sheep treated with acetazolamide or amiloride. This apparent difference was attributable to one sheep in the furosemide cohort with high serum creatinine, and thus presumably some level of pre‐existing kidney disease. Importantly, the pattern of effects of furosemide was similar, regardless of whether this sheep was included in the analysis or not.

Using the same methodology to directly assess intra‐renal tissue oxygenation in non‐anaesthetised sheep as used in the current study, Iguchi and colleagues showed that furosemide treatment at the same dosage to that used in our current study significantly increased renal medullary tissue $P_{O_{2}}$ in a clinically relevant sheep model of established septic acute kidney injury (Iguchi et al., 2019). However, furosemide treatment did not alter renal cortical tissue $P_{O_{2}}$ in ovine septic acute kidney injury (Iguchi et al., 2019). The ability of furosemide to improve renal medullary oxygenation was also inferred from observations in human septic shock from measurement of bladder urinary $P_{O_{2}}$ as an index of medullary tissue $P_{O_{2}}$ (Osawa et al., 2019). These observations reinforce our proposition that the effect of furosemide on intra‐renal tissue oxygenation is likely to be different in health than in the setting of pre‐existing kidney disease.

We were unable to detect a significant increase in urine flow after administration of a clinically relevant dose of amiloride. Nevertheless, it is noteworthy that this dose of amiloride had no apparent effect on renal oxygenation in a healthy sheep, at least within the 8 h experimental period. To the best of our knowledge, data on the effects of amiloride on tissue oxygenation, using either direct or indirect methods, are scant. However, simulations derived from a mathematical model of solute transport along the nephron indicate that inhibition of the epithelial sodium channel, such as that by amiloride, has little effect on sodium delivery along the entire nephron length (Layton et al., 2016). Inhibition of active transport via the epithelial sodium channel may in turn decrease paracellular reabsorption of sodium by ∼40% (Pei et al., 2016). The latter would not directly affect oxygen utilisation for tubular sodium transport because paracellular reabsorption is a passive process (Layton et al., 2016).

Strengths of our current study include the use of healthy unanaesthetised animals, a direct method for measurement of tissue $P_{O_{2}}$, and doses of acetazolamide, furosemide and amiloride that are relevant to clinical human medicine. Limitations include the fact that our study did not include animals with disorders for which these agents would be indicated (e.g., hypertension, acute kidney injury, chronic kidney disease or heart failure). These conditions are often characterised by factors that can contribute to renal hypoxia, such as renal fibrosis, endothelial dysfunction, anaemia and so on (Ow et al., 2018). Thus, the effects of diuretics on renal tissue oxygenation under these conditions may differ from those in healthy unanaesthetised sheep. It is also important to acknowledge that in clinical medicine, diuretics are often (although not exclusively) administered chronically (e.g., for hypertension or heart failure) or as intermittent boluses (e.g., for patients with fluid retention in the intensive care unit). Our findings and conclusions regarding the effects of acute administration of diuretics in healthy sheep cannot be generalised to such clinical scenarios. Additionally, because plasma and urinary creatinine and sodium concentrations were measured only on the baseline sample and the samples collected during the fourth and eighth hour after treatment had commenced, we potentially missed the peak diuretic period when the greatest change of urinary excretion of sodium likely occurred. Indeed, we have previously reported significant increases in urinary output, urinary sodium excretion and fractional sodium excretion in healthy and septic sheep by 1 h after furosemide treatment had commenced (Iguchi et al., 2019; Lankadeva et al., 2014). We also acknowledge that our inability to detect effects of amiloride on urine flow during the 8 h experimental period limits our ability to draw inferences about the impact of this agent on renal oxygenation. It is noteworthy that when the same clinical dose of amiloride (20 mg) was given as a slow intravenous infusion over 24 h in healthy female sheep (Reid et al., 1990), it evoked significant increases in urinary sodium excretion. This observation provides some confidence that this dose of amiloride is likely to be sufficient to inhibit the epithelial sodium channels in the distal collecting tubules.

Lastly, there have been recent reports of sex differences in renal physiology, including the abundance and localisation of transporters in the kidney (McDonough & Layton, 2023). For example, female rats have lesser abundance and activity of NHE3 transporters and aquaporin‐1 in the proximal tubules (Veiras et al., 2017). Consequentially, bicarbonate and sodium reabsorption at the proximal tubule is less in female than male rats (Veiras et al., 2017). Therefore, there is a likelihood of greater demand for filtrate reabsorbed in the distal portion of the nephrons in female than male rats. This is evidenced by ∼20%–40% greater abundance of NKCC2 transporters at the loop of Henle (Veiras et al., 2017) and two‐fold greater abundance of Na^+^–K^+^–Cl^−^ cotransporters, phosphorylated Na^+^–K^+^–Cl^−^ cotransporters and epithelial sodium channels at the distal collecting ducts in female than male rats (Veiras et al., 2017). Therefore, the magnitude of the effects of diuretics on renal tissue oxygenation likely differ between the sexes and our findings are likely to be more applicable to females.

### 4.1 Experimental limitation

In this study, we examined the effects of three major classes of diuretic drugs, at clinically relevant doses, on renal and intra‐renal perfusion and oxygenation and bladder urinary oxygenation and kidney function in non‐anaesthetised healthy sheep. However, a major shortcoming of the experimental design, which limited our ability to adequately delineate the time course of alterations in renal sodium reabsorption evoked by furosemide, was the collection of plasma and urine samples outside the period of peak diuresis (at 1 h post furosemide infusion). Nevertheless, continuous measurement of renal cortical and medullary tissue oxygenation at hourly intervals after furosemide administration demonstrated that renal cortical and medullary and bladder urinary oxygen levels did not increase even during this peak diuretic period (Iguchi et al., 2019). An additional limitation relates to the fact that local renal tissue oxygenation is determined by the balance between local tissue oxygen delivery and consumption. Although we can assess local tissue oxygen delivery, at least to some extent, by consideration of blood oxygen content and local tissue perfusion, we are limited by the unavailability of techniques to measure local tissue oxygen consumption in non‐anaesthetised sheep. In the context of the current study, lack of this measurement prevents us from drawing definitive conclusions regarding the determinants of the effects of furosemide, acetazolamide and amiloride on renal cortical and medullary oxygenation. Therefore, future studies are warranted to investigate local tissue oxygen consumption along specific nephron segments during peak period of diuresis and natriuresis to better understand the effects of clinically used diuretics.

In conclusion, our current findings indicate little impact of clinically relevant doses of acetazolamide, furosemide or amiloride on renal cortical or medullary tissue oxygenation at least within the 8 h post‐diuretic time frame. These observations provide some level of assurance that these agents are unlikely to induce or exacerbate renal hypoxia in association with their diuretic effects. However, our findings also do not support the hypothesis that these agents can improve renal oxygenation, at least in the settings of no pre‐existing kidney disease. Indeed, rebound hypoxia after the dissipation of furosemide‐induced diuresis could contraindicate its use in some circumstances.

## AUTHOR CONTRIBUTIONS

Connie P. C. Ow, Nobuki Okazaki, Naoya Iguchi, Rachel M. Peiris, Sally G. Hood were involved in acquisition, analysis, or interpretation of data. Roger G. Evans, Clive N. May, Rinaldo Bellomo and Yugeesh R. Lankadeva were involved in conception and design of the work. All authors were involved in drafting of the work and revising it critically for important intellectual content. All authors approved the final version of the manuscript and agree to be accountable for all aspects of the work in ensuring that questions related to the accuracy or integrity of any part of the work are appropriately investigated and resolved. All persons designated as authors qualify for authorship, and all those who qualify for authorship are listed.

## CONFLICT OF INTEREST

The authors declare no conflicts of interest.

## Data Availability

The data that support the findings of this study are available from the authors upon reasonable requests.

## References

[eph13523-bib-0001] Abdeen, A. , Sonoda, H. , Oshikawa, S. , Hoshino, Y. , Kondo, H. , & Ikeda, M. (2016). Acetazolamide enhances the release of urinary exosomal aquaporin‐1. Nephrology, Dialysis, Transplantation, 31(10), 1623–1632. 10.1093/ndt/gfw033 27190370

[eph13523-bib-0002] Bailey, M. A. , Giebisch, G. , Abbiati, A. , Aronson, P. S. , Gawensin, L. R. , Shull, G. E. , & Wang, T. (2004). NHE2‐mediated bicarbonate reasborption in the distal tubule of NHE3 null mice. The Journal of Physiology, 561(3), 765–775. 15604231 10.1113/jphysiol.2004.074716 PMC1665379

[eph13523-bib-0003] Bouquet, M. , Passmore, M. R. , See Hoe, L. E. S. , Tung, J. P. , Simonova, G. , Boon, A. C. , & Fraser, J. F. (2020). Development and validation of ELISAs for the quantitation of interleukin (IL)‐1β, IL‐6, IL‐8 and IL‐10 in ovine plasma. Journal of Immunological Methods, 486, 112835. 32828792 10.1016/j.jim.2020.112835

[eph13523-bib-0004] Brezis, M. , Agmon, Y. , & Epstein, F. H. (1994). Determinants of intrarenal oxygenation. I. Effects of diuretics. American Journal of Physiology, 267, F1059–F1062. 7810692 10.1152/ajprenal.1994.267.6.F1059

[eph13523-bib-0005] Castrop, H. , & Schießl, I. M. (2014). Physiology and pathophysiology of the renal Na‐K‐2Cl cotransporter (NKCC2). American Journal of Physiology‐Renal Physiology, 307(9), F991–F1002. 25186299 10.1152/ajprenal.00432.2014

[eph13523-bib-0006] Epstein, F. H. & , & Prasad, P. (2000). Effects of furosemide on medullary oxygenation in younger and older subjects. Kidney International, 57(5), 2080–2083. 10792627 10.1046/j.1523-1755.2000.00057.x

[eph13523-bib-0007] Evans, R. G. , Gardiner, B. S. , Smith, D. W. , & O'Connor, P. M. (2008). Methods for studying the physiology of kidney oxygenation. Clinical and Experimental Pharmacology & Physiology, 35(12), 1405–1412. 18983577 10.1111/j.1440-1681.2008.05063.x

[eph13523-bib-0008] Felker, G. M. , Ellison, D. H. , Mullens, W. , Cox, Z. , & Testani, J. M. (2020). Diuretic therapy for patients with heart failure: JACC State‐of‐the‐Art Review. Journal of the American College of Cardiology, 75(10), 1178–1195. 32164892 10.1016/j.jacc.2019.12.059

[eph13523-bib-0009] Fine, L. G. & , & Norman, J. T. (2008). Chronic hypoxia as a mechanism of progression of chronic kidney diseases: From hypothesis to novel therapeutics. Kidney International, 74(7), 867–872. 18633339 10.1038/ki.2008.350

[eph13523-bib-0010] Haddock, B. , Larsson, H. B. W. , Francis, S. & , & Andersen, U. B. (2019). Human renal response to furosemide: Simultaneous oxygenation and perfusion measurements in cortex and medulla. Acta Physiologica, 227(1), e13292. 31046189 10.1111/apha.13292 PMC6767552

[eph13523-bib-0011] Huang, X. , Mees, E. D. , Vos, P. , Hamza, S. , & Braam, B. (2016). Everything we always wanted to know about furosemide but were afraid to ask. American Journal of Physiology‐Renal Physiology, 310(10), F958–F971. 26911852 10.1152/ajprenal.00476.2015

[eph13523-bib-0012] Iguchi, N. , Lankadeva, Y. R. , Mori, T. A. , Osawa, E. A. , Cutuli, S. L. , Evans, R. G. , Bellomo, R. , & May, C. N. (2019). Furosemide reverses medullary tissue hypoxia in ovine septic acute kidney injury. American Journal of Physiology‐Regulatory, Integrative and Comparative Physiology, 317(2), R232–R239. 31141418 10.1152/ajpregu.00371.2018

[eph13523-bib-0013] Kiil, F. (1977). Renal energy metabolism and regulation of sodium reabsorption. Kidney International, 11(3), 153–160. 139494 10.1038/ki.1977.23

[eph13523-bib-0014] Krishnan, D. , Liu, L. , Casey, J. R. , Cordat, E. , & Alexander, R. T. (2015). Carbonic anhydrase II binds to and increases the activity of the epithelial sodium‐proton exchanger, NHE3. American Journal of Physiology‐Renal Physiology, 309(4), F383–F392. 26041446 10.1152/ajprenal.00464.2014 PMC4959884

[eph13523-bib-0015] Lal, H. , Singh, P. , Ponmalai, K. , Prasad, R. , Singh, S. P. , Yadav, P. , Singh, A. , Bhadauria, D. , Kumar, S. , Agarwal, V. , & Mishra, P. (2022). Role of blood oxygen level‐dependent magnetic resonance imaging in studying renal oxygenation changes in renal artery stenosis. Abdominal Radiology (NY), 47(3), 1112–1123. 10.1007/s00261-022-03408-5 35059812

[eph13523-bib-0016] Lankadeva, Y. R. , Kosaka, J. , Evans, R. G. , & May, C. N. (2018). An ovine model for studying the pathophysiology of septic acute kidney injury. In B. Tharakan (Ed.), Methods in molecular biology, Clifton, N.J. : Humana Press (Vol. 1717, pp. 207–218). 29468594 10.1007/978-1-4939-7526-6_16

[eph13523-bib-0017] Lankadeva, Y. R. , Ma, S. , Iguchi, N. , Evans, R. G. , Hood, S. G. , Farmer, D. G. S. , Bailey, S. R. , Bellomo, R. , & May, C. N (2019). Dexmedetomidine reduces norepinephrine requirements and preserves renal oxygenation and function in ovine septic acute kidney injury. Kidney International, 96(5), 1150–1161. 31530477 10.1016/j.kint.2019.06.013

[eph13523-bib-0018] Lankadeva, Y. R. , May, C. N. , McKinley, M. J. , Neeland, M. R. , Ma, S. , Hocking, D. M. , Robins‐Browne, R. , Bedoui, S. , Farmer, D. G. S. , Bailey, S. R. , Martelli, D. , & McAllen, R. M. (2020). Sympathetic nerves control bacterial clearance. Scientific Reports, 10(1), 15009. 32929135 10.1038/s41598-020-72008-4 PMC7490383

[eph13523-bib-0019] Lankadeva, Y. R. , Singh, R. R. , Hilliard, L. M. , Moritz, K. M. , & Denton, K. M. (2014). Impaired ability to modulate glomerular filtration rate in aged female sheep following fetal uninephrectomy. Physiological Reports, 2(1), e00208. 24744887 10.1002/phy2.208 PMC3967691

[eph13523-bib-0020] Layton, A. T. , Laghmanu, K. , Vallon, V. , & Edwards, A. (2016). Solute transport and oxygen consumption along the nephrons: Effects of Na^+^ transport inhibitors. American Journal of Physiology‐Renal Physiology, 311(6), F1217–F1229. 27707706 10.1152/ajprenal.00294.2016 PMC5210208

[eph13523-bib-0021] Layton, A. T. , Vallon, V. , & Edwards, A. (2015). Modeling oxygen consumption in the proximal tubule: Effects of NHE and SGLT2 inhibition. American Journal of Physiology‐Renal Physiology, 308(12), F1343–F1357. 25855513 10.1152/ajprenal.00007.2015 PMC4469883

[eph13523-bib-0022] Lee, S.‐K. , Lee, J. , Hang, S. , Lee, E. , Jeon, C.‐Y. , Lim, K.‐S. , Jin, Y. B. , & Choi, J. (2021). Quantification of renal T2 relaxation rate by use of blood oxygen level‐dependent magnetic resonance imaging before and after furosemide administration in healthy Beagles. American Journal of Veterinary Research, 82(11), 880–889. 34669496 10.2460/ajvr.82.11.880

[eph13523-bib-0023] Ludbrook, J. (1994). Repeated measures and multiple comparisons in cardiovascular research. Cardiovascular Research, 28(3), 303–311. 8174149 10.1093/cvr/28.3.303

[eph13523-bib-0024] McCoy, I. E. , Chertow, G. M. , & Chang, T. I. (2019). Patterns of diuretic use in the intensive care unit. PLoS ONE, 14(5), e0217911. 31150512 10.1371/journal.pone.0217911 PMC6544280

[eph13523-bib-0025] McDonough, A. A. , & Layton, A. T. (2023). Sex differences in renal electrolyte transport. Current Opinion in Nephrology and Hypertension, 32(5), 467–475. 37382185 10.1097/MNH.0000000000000909 PMC10526720

[eph13523-bib-0026] Mutchler, S. M. , & Kleyman, T. R. (2019). New insights regarding epithelial Na^+^ channel regulation and its role in the kidney, immune system and vasculature. Current Opinion in Nephrology and Hypertension, 28(2), 113–119. 30585851 10.1097/MNH.0000000000000479 PMC6349474

[eph13523-bib-0027] Neeland, M. R. , Elhay, M. J. , Nathanielsz, J. , Meeusen, E. N. T. , & De Veer, M. J. (2014). Incorporation of CpG into a liposomal vaccine formulation increases the maturation of antigen‐loaded dendritic cells and monocytes to improve local and systemic immunity. Journal of Immunology, 192(8), 3666–3675. 10.4049/jimmunol.1303014 24646740

[eph13523-bib-0028] Orlov, S. N. , Kolstsova, S. V. , Kapilevich, L. V. , Gusakova, S. V. , & Dulin, N. O. (2015). NKCC1 and NKCC2: The pathogenetic role of cation‐chloride cotransporters in hypertension. Genes & Diseases, 2(2), 186–196. 26114157 10.1016/j.gendis.2015.02.007 PMC4477834

[eph13523-bib-0029] Osawa, E. A. , Cutuli, S. L. , Bitker, L. , Canet, E. , Cioccari, L. , Iguchi, N. , Lankadeva, Y. R. , Eastwood, G. M. , Evans, R. G. , May, C. N. , & Bellomo, R. (2019). Effect of furosemide on urinary oxygenation in patients with septic shock. Blood Purification, 48(4), 336–345. 31336370 10.1159/000501512

[eph13523-bib-0030] Ow, C. P. C. , Ngo, J. P. , Ullah, M. M. , Hilliard, L. M. , & Evans, R. G. (2018). Renal hypoxia in kidney disease: Cause or consequence? Acta Physiologica, 222(4), e12999. 29159875 10.1111/apha.12999

[eph13523-bib-0031] Pedersen, M. , Vajda, Z. , Stødkilde‐Jørgensen, H. , Nielsen, S. , & Frøkiaer, J. (2007). Furosemide increases water content in renal tissue. American Journal of Physiology‐Renal Physiology, 292(5), F1645–F1651. 17264309 10.1152/ajprenal.00060.2006

[eph13523-bib-0032] Pei, L. , Solis, G. , Bguyen, M. T. X. , Kamat, N. , Magenheimer, L. , Zhuo, M. , Li, J. , Curry, J. , McDonough, A. A. , Fields, T. A. , Welch, W. J. , & Yu, A. S. L. (2016). Paracellular epithelial sodium transport maximizes energy efficiency in the kidney. Journal of Clinical Investigation, 126(7), 2509–2518. 27214555 10.1172/JCI83942 PMC4922683

[eph13523-bib-0033] Peiris, R. M. , May, C. N. , Booth, L. C. , McAllen, R. M. , McKinley, M. J. , Hood, S. G. , Martelli, D. , Bellomo, R. , & Lankadeva, Y. R. (2023). Splanchnic sympathetic nerve denervation improves bacterial clearance and clinical recovery in established ovine Gram‐negative bacteremia. Intensive Care Medicine Experimental, 11(1), 53. 37535121 10.1186/s40635-023-00530-6 PMC10400745

[eph13523-bib-0034] Percie du Sert, N. , Hurst, V. , Ahluwalia, A. , Alam, S. , Avey, M. T. , Baker, M. , Browne, W. J. , Clark, A. , Cuthill, I. C. , Dirnagl, U. , Emerson, M. , Garner, P. , Holgate, S. T. , Howells, D. W. , Karp, N. A. , Lazic, S. E. , Lidster, K. , MacCallum, C. J. , Macleod, M. , … Würbel, H. (2020). The ARRIVE guidelines 2.0: Updated guidelines for reporting animal research. PLoS Biology, 18(7), e3000410. 32663219 10.1371/journal.pbio.3000410 PMC7360023

[eph13523-bib-0035] Prasad, P. V. , Edelman, R. R. , & Epstein, F. H. (1996). Noninvasive evaluation of intrarenal oxygenation with BOLD MRI. Circulation, 94(12), 3271–3275. 8989140 10.1161/01.cir.94.12.3271

[eph13523-bib-0036] Priatna, A. , Epstein, F. H. , Spokes, K. , & Prasad, P. V. (1999). Evaluation of changes in intrarenal oxygenation in rats using multiple gradient‐recalled echo (mGRE) sequence. Journal of Magnetic Resonance Imaging, 9(6), 842–846. 10373033 10.1002/(sici)1522-2586(199906)9:6<842::aid-jmri12>3.0.co;2-v PMC2914481

[eph13523-bib-0037] Purkerson, J. M. , & Schwartz, G. J. (2007). The role of carbonic anhydrases in renal physiology. Kidney International, 71(2), 103–115. 17164835 10.1038/sj.ki.5002020

[eph13523-bib-0038] Reid, A. F. , Coghlan, J. P. , Whitworth, J. A. , & Scoggins, B. A. (1990). Amiloride blocks the onset of ACTH‐induced hypertension in sheep. American Journal of Hypertension, 3(10_Pt_1), 775–781. 2171563 10.1093/ajh/3.10.775

[eph13523-bib-0039] Roush, G. C. , Kaur, R. , & Ernst, M. E. (2014). Diuretics: A review and update. Journal of Cardiovascular Pharmacology and Therapeutics, 19(1), 5–13. 24243991 10.1177/1074248413497257

[eph13523-bib-0040] Sica, D. A. (2012). Diuretic use in renal disease. Nature Reviews Nephrology, 8(2), 100–109. 10.1038/nrneph.2011.175 22183505

[eph13523-bib-0041] Sun, Q. , & Sever, P. (2020). Amiloride: A review. Journal of the Renin‐Angiotensin‐Aldosterone System, 21(4), 1–9. 10.1177/1470320320975893 PMC7691917 33234024

[eph13523-bib-0042] Tanaka, S. , Tanaka, T. , & Nangaku, M. (2014). Hypoxia as a key player in the AKI‐to‐CKD transition. American Journal of Physiology‐Renal Physiology, 307(11), F1187–F1195. 25350978 10.1152/ajprenal.00425.2014

[eph13523-bib-0043] Ullah, M. M. , & Basile, D. P. (2019). Role of renal hypoxia in the progression from acute kidney injury to chronic kidney disease. Seminars in Nephrology, 39(6), 567–580. 31836039 10.1016/j.semnephrol.2019.10.006 PMC6917038

[eph13523-bib-0044] Van Berkel, M. A. , & Elefritz, J. L. (2018). Evaluating off‐label use of acetazolamide. American Journal of Health‐System Pharmacy, 75(8), 524–531. 29626002 10.2146/ajhp170279

[eph13523-bib-0045] Van Wart, S. A. , Shoaf, S. E. , Mallikaarjun, S. , & Mager, D. E. (2014). Population based meta‐analysis of furosemide pharmacokinetics. Biopharmaceutics & Drug Disposition, 35(2), 119–133. 24151207 10.1002/bdd.1874

[eph13523-bib-0046] Veiras, L. C. , Girardi, A. C. C. , Curry, J. , Pei, L. , Ralph, D. L. , Tran, A. , Castelo‐Branco, R. C. , Pastor‐Soler, N. , Arranz, C. T. , Yu, A. S. L. , & McDonough, A. A. (2017). Sexual dimorphism pattern of renal transporters and electrolyte homeostasis. Journal of the American Society of Nephrology, 28(12), 3504–3517. 28774999 10.1681/ASN.2017030295 PMC5698077

[eph13523-bib-0047] Warner, L. , Glockner, J. F. , Woollard, J. , Textor, S. C. , Romero, J. C. , & Leman, L. O. (2011). Determinations of renal cortical and medullary oxygenation using BOLD magnetic resonance imaging and selective diuretics. Investigative Radiology, 46(1), 41–47. 20856128 10.1097/RLI.0b013e3181f0213f PMC3006042

[eph13523-bib-0048] Zhang, J. , An, Y. , Gao, J. , Han, J. , Pan, X. , Pan, Y. , Tie, L. , & Li, X. (2012). Aquaporin‐1 translocation and degradation mediates the water transportation mechanism of acetazolamide. PLoS ONE, 7(9), e45976. 23029347 10.1371/journal.pone.0045976 PMC3448731

